# Deep 16S rRNA Pyrosequencing Reveals a Bacterial Community Associated with Banana *Fusarium* Wilt Disease Suppression Induced by Bio-Organic Fertilizer Application

**DOI:** 10.1371/journal.pone.0098420

**Published:** 2014-05-28

**Authors:** Zongzhuan Shen, Dongsheng Wang, Yunze Ruan, Chao Xue, Jian Zhang, Rong Li, Qirong Shen

**Affiliations:** 1 National Engineering Research Center for Organic-based Fertilizers, Key Laboratory of Plant Nutrition and Fertilization in Low-Middle Reaches of the Yangtze River, Ministry of Agriculture, Jiangsu Key Lab and Engineering Center for Solid Organic Waste Utilization, Jiangsu Collaborative Innovation Center for Solid Organic Waste Resource Utilization, Nanjing Agricultural University, Nanjing, China; 2 Hainan key Laboratory for Sustainable Utilization of Tropical Bio-resources, College of Agriculture, Hainan University, Haikou, China; 3 Nanjing Institute of Vegetable Science, Nanjing, China; Graz University of Technology (TU Graz), Austria

## Abstract

Our previous work demonstrated that application of a bio-organic fertilizer (BIO) to a banana mono-culture orchard with serious *Fusarium* wilt disease effectively decreased the number of soil *Fusarium* sp. and controlled the soil-borne disease. Because bacteria are an abundant and diverse group of soil organisms that responds to soil health, deep 16 S rRNA pyrosequencing was employed to characterize the composition of the bacterial community to investigate how it responded to BIO or the application of other common composts and to explore the potential correlation between bacterial community, BIO application and *Fusarium* wilt disease suppression. After basal quality control, 137,646 sequences and 9,388 operational taxonomic units (OTUs) were obtained from the 15 soil samples. *Proteobacteria*, *Acidobacteria*, *Bacteroidetes*, *Gemmatimonadetes* and *Actinobacteria* were the most frequent phyla and comprised up to 75.3% of the total sequences. Compared to the other soil samples, BIO-treated soil revealed higher abundances of *Gemmatimonadetes* and *Acidobacteria*, while *Bacteroidetes* were found in lower abundance. Meanwhile, on genus level, higher abundances compared to other treatments were observed for *Gemmatimonas* and *Gp4*. Correlation and redundancy analysis showed that the abundance of *Gemmatimonas* and *Sphingomonas* and the soil total nitrogen and ammonium nitrogen content were higher after BIO application, and they were all positively correlated with disease suppression. Cumulatively, the reduced *Fusarium* wilt disease incidence that was seen after BIO was applied for 1-year might be attributed to the general suppression based on a shift within the bacteria soil community, including specific enrichment of *Gemmatimonas* and *Sphingomonas*.

## Introduction

Banana *Fusarium* wilt disease, which is caused by *Fusarium oxysporum* f. sp. *cubense* race 4 (FOC) and reported to be the most limiting factor in banana production worldwide, has spread quickly in *Cavendish*-production areas since 1996, and it affects approximately 90% of the banana industry in China [1]–[3]. Among the managements for controlling the disease, such as crop rotation, biocontrol, application of chemical fungicides and cropping of resistant banana cultivars [4]–[8], biocontrol is the most promising technique for disease prevention because of owning the advantages of environmental protection, safety, high economic benefits and longevity at the same time [9]. However, direct inoculation of functional microorganisms into the soil without a suitable organic substrate cannot be expected to be successful due to the absence of nutrients [10]. Many reports have demonstrated that biocontrol agents combined with organic materials to create novel bio-organic fertilizers (BIOs) can enhance the suppression of *Fusarium* wilt disease in the soil by ameliorating the structure of the microbial community [11]–[14].

The composition of the soil microbial community and induced changes caused by its amendment, provide useful information on soil health and quality [15]. Maintaining biodiversity of soil microbes is crucial to soil health because a decrease in soil microbial diversity is responsible for the development of soil-borne diseases [16]. Determining the responses of soil bacterial communities to different organic amendments is particularly important because the bacterial community is one of the main components that determine soil health and is believed to be one of the main drivers in disease suppression [17]. Despite the known key roles of bacteria in soil health and the significant change in soil bacterial composition and activity after BIO application, information regarding the variation of soil bacterial communities that are affected by different organic amendments is still lacking. More importantly, understanding soil microbial community structure shifts following implementation of various organic amendments is an important component when selecting fertilizer types to improve soil function and health.

As described in our previous work, *Fusarium* wilt disease was more effectively controlled by a 1-year application of BIO than by the other composts in a field experiment [12]. In that study, the effects of different types of composts on soil bacterial communities were mainly assessed using traditional PCR-DGGE fingerprinting and culture-dependent methods. Taking into account the large size of the bacterial community and the heterogeneity of the soils, only a tiny fraction of the bacterial diversity was unraveled by that study. Recently, pyrosequencing of 16 S rRNA gene fragments has been applied for in-depth analysis of soil bacterial communities [18], [19]. This method could provide a large number of parallel reads to characterize the unseen majority of the soil microbial community and offer an opportunity to achieve a high throughput and deeper insight into the effects of different types of composts on soil bacterial communities [20], thus it is an improvement over previous fingerprinting techniques, such as PCR-DGGE or T-RFLP, which are not entirely specific and do not result in many sequences [15].

We used a deep 16 S rRNA pyrosequencing approach to further investigate how the soil bacteria community responded to the application of BIO or other common composts and to explore the potential correlation between bacterial community, BIO application and *Fusarium* wilt disease suppression. This study was the first to provide information on the banana soil bacterial community in a single soil type that was exposed to different organic amendments using deep 16 S rRNA pyrosequencing. Therefore, the aims of this study were to answer the following questions: (1) Does the soil bacteria community that is amended with BIO differ from that exposed to other common composts? (2) Does the *Fusarium* wilt disease incidence correlate with the bacterial community? (3) Does the disease suppression after BIO application correlate with the physicochemical properties of the soil?

## Materials and Methods

### Ethics statement

Our study was carried out on the farmers' land (18°23′ N, 108°44′ E) with property rights in China (1996-2035) and farmer Yusheng Li should be contacted for future permissions. No specific permits were required for the described field studies and the locations are not protected. The field studied did not involve endangered or protected species.

### Field experiment

Five treatments were established as randomized, complete block designs with three replicates at the “Wan Zhong” banana orchard in Hainan, China and included a general operation control (GCK) and soil that was amended with four different types of organic amendments: bio-organic fertilizer (BIO), cattle manure compost (CM), Chinese medicine residue compost (CMR) and pig manure compost (PM). And each replicate was planted with 170 banana tissue culture plantlets (*Musa acuminate* AAA *Cavendish* cv. Brazil) with an area of 667 m^2^. Worthy to notify, the bio-organic fertilizer (BIO) contained a biocontrol agent *Bacillus* sp. and was prepared by a solid fermentation method according to Chen et al. [21]. The orchard has been continuously cropped banana for more than 10 years and was abandoned by farmers to growing banana for high *Fusarium* wilt disease incidence (50%). The detailed information regarding the field experiment setting and amendments were described in our previous report [12].

### Soil sample collection and DNA extraction

The soil sample collection and DNA extraction methods were described in detail as supplementary information to our previous study [12]. Five individual, healthy banana trees that were at least 5 m apart in each treatment plot were randomly selected for sample collection, and the collected soil samples from each tree were mixed as a composite soil sample for each replicate plot. For each tree, composite soil from 4 random sites of the trunk base was collected using a 25-mm soil auger at a depth of 20 cm. All soil samples were transported to the laboratory and stored at −70°C for subsequent DNA extraction after sifting through a 2-mm sieve. Total soil DNA was extracted using PowerSoil DNA Isolation Kits (MoBio Laboratories Inc., Carlsbad, USA) according to the manufacturer's protocol. The concentration and quality (ratio of A260/A280) of the DNA were determined using a spectrophotometer (NanoDrop 2000, ThermoScientific, USA).

### Polymerase chain reaction amplification and deep 16 S rRNA pyrosequencing

PCR reactions for each sample were performed in triplicate (including two negative control reactions) with 2 µM of each primer, 0.25 µM of dNTPs, 4 µL of 5 × FastPfu Buffer, 1 U of FastPfu DNA polymerase (2.5 U/µL, TransGen Biotech Co., Ltd., Beijing, China) and approximately 20 ng of soil DNA template at a final volume of 20 µL. The forward primer consisted of the 25-bp 454 adapter A, 2-bp linker A and 15-bp universal bacterial primer 27F [22], and the reverse primer consisted of the 25-bp 454 adapter B, 2-bp linker B, a 10-bp barcode and the 19-bp universal bacterial primer 533R [23]. Detailed information regarding the primer sequence is shown in Table S1. These primers target an approximately 500-bp region of the 16 S rRNA gene that contains variable regions 1 to 3 (V1–V3), which is well-suited for accurate phylogenetic placement of bacterial sequences [24].

Amplifications were performed using an Eppendorf Mastercycler thermocycler (Eppendorf North America, Hauppauge, NY) with the following temperature program: an initial denaturation step of 95°C for 4 min, followed by 25 cycles of denaturation at 95°C for 30 s, annealing at 55°C for 30 s, extension at 72°C for 30 s and a final elongation at 72°C for 5 min. PCR amplicon libraries were purified from a 1.2% agarose gel and quantified using the PicoGreen dsDNA reagent (Promega, USA). Equal amplicons from each sample were then pooled in equimolar concentrations into a single aliquot. After cleaning, precipitating, and re-suspending the amplicons in nuclease-free water, an emPCR was carried out to attach the single strands onto beads for further 454 pyrosequenicng. Pyrosequencing was performed on a Roche 454 GS-FLX Titanium System at Majorbio Bio-pharm Technology Co., Ltd (Shanghai, China).

### Bioinformatic analysis

After pyrosequencing, raw sequences were analyzed using the Mothur software following the Schloss standard operating procedure [25]. Briefly, sequences with a minimum flow length of 450 flows were denoised using the Mothur-based reimplementation of the PyroNoise algorithm with the default parameters [26]. Sequences with more than 1 mismatch to the barcode, 2 mismatches to the primer, any ambiguous base call, homopolymers longer than 8 bases and reads shorter than 250 bp were eliminated, and the filtered sequences were then trimmed and assigned to soil samples based on unique 10-base barcodes. After removing the barcode and primer sequences, the unique sequences were aligned against the Silva bacteria database [27]. After screening, filtering, preclustering, and chimera removal, the retained sequences were used to build a distance matrix with a distance threshold of 0.2. Using the average neighbor algorithm with a cut-off of 97% similarity, bacterial sequences were clustered to operational taxonomic units (OTU), and the representative sequence for each OTU was picked and classified using a Ribosomal Database Project naive Bayesian rRNA classifier with a confidence threshold of 80% [28]. Lastly, the resulting matches for each set of sequence data were summarized at various levels of taxonomic hierarchal structure (e.g., phylum and genera). All raw sequences have been deposited in DDBJ SRA under the accession number DRA001282.

To correct for sampling effects, we used a randomly selected subset of 7,817 sequences per sample to further analyze the richness and diversity of the bacterial community. All analyses were based on the OTU clusters with a cut-off of 3% dissimilarity. The richness index of the Chao1 estimator (Chao1) [29] and the abundance-based Coverage estimator (ACE) [30] was calculated to estimate the number of observed OTUs that were present in the sampling assemblage. The diversity within each individual sample was estimated using the nonparametric Shannon diversity index [31]. Good's nonparametric Coverage estimator was used to estimate the percentage of the total species that were sequenced in each sample [32], and a rarefaction curve generated using the Mothur software was used to compare the relative levels of bacterial OTU diversity across all soil samples.

To compare bacterial community structures across all samples, a heat map based on the abundant phyla were performed in R (Version 3.0.2) with the gplots package [33], [34], and principal coordinates analysis (PCoA) based on the OTU composition was performed using the Mothur software. To examine the relationship between the frequencies of abundant phyla, samples and environmental variables, redundancy analysis (RDA) was carried out using CANOCO for Windows [35].

### Statistical analysis

The relationships between the selected taxonomy group (abundant phyla or genera) or bacterial community indices (Chao1, ACE and Shannon) and *Fusarium* wilt disease incidence (DI) were calculated using the SPSS 13.0 software program. For all parameters, data were compared using a one-way analysis of variance (ANOVA) at the end of each bioassay. Mean comparison was performed using Fisher's least significant difference test (LSD) and the Duncan multiple range test with a significance level of p<0.05.

## Results

After filtering the reads based on basal quality control, 137,646 sequences with an average length of 254 bases were obtained from 15 soil samples when using Mothur flowgrams strategy to analyze sequences. The number of high-quality sequences per sample varied from 7,817 to 11,234 (Table 1). Based on 97% species similarity, in total 9,388 OTUs were found, and 12,845 sequences (9.3% of the total sequences) were returned as unclassified.

**Table 1:** Good quality sequences that were used to further analysis after basic quality control for treatments: bio-organic fertilizer (BIO), cattle manure compost (CM), Chinese medicine residue compost (CMR), general operation control (GCK) and pig manure compost (PM).

Treatments	Good quality sequences
BIO1	9,382
BIO2	9,666
BIO3	7,817
CM1	9,937
CM2	8,521
CM3	8,459
CMR1	8,736
CMR2	9,280
CMR3	8,614
GCK1	8,192
GCK2	8,473
GCK3	11,234
PM1	8,695
PM2	11,185
PM3	9,455
Total	137,646

### Bacterial community composition

**Figure 1:**
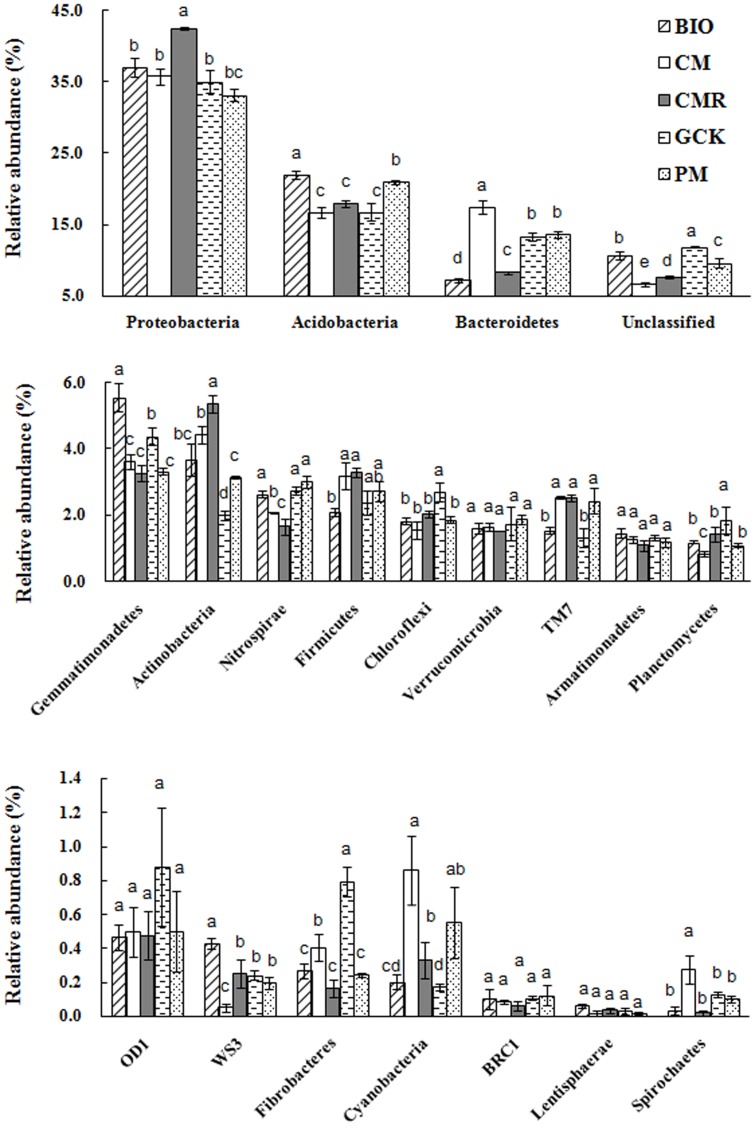
The relative abundance of the phyla for treatments with bio-organic fertilizer (BIO), cattle manure compost (CM), Chinese medicine residue compost (CMR), general operation control (GCK) and pig manure compost (PM). Bars represent the standard error of the three replicates and different letters above each phylum indicate significantly difference at 0.05 probability level according to the Duncan test.

As shown in Fig. 1, although the phyla compositions of the different soil samples were similar, some obvious variations in the relative abundances of phyla between different fertilizer treatments were still observed. The classified sequences for each sample were affiliated with 19 bacterial phyla, and the remaining sequences were unclassified. The most abundant phyla of *Proteobacteria*, *Acidobacteria* and *Bacteroidetes* were found in all treatments at a relative abundance of approximately 35%, 15% and 10%, respectively, and 9 phyla (*Actinobacteria*, *Gemmatimonadetes*, *Nitrospirae*, *Firmicutes*, *Chloroflexi*, *Verrucomicrobia*, *TM7*, *Armatimonadetes* and *Planctomycetes*) were found in all samples at a relative abundance of higher than 1%, but lower than 6%, with some obvious variations. The relative abundances of *Acidobacteria* and *Gemmatimonadetes* were highest, while those of *Bacteroidetes* were lowest, in the BIO-treated soil sample compared with the other treatments (CM, CMR, GCK and PM).

The most abundant classified genera (>1%) for each treatment are shown in Table 2, which shows 12, 16, 14, 12 and 15 most frequently classified genera for the BIO, CM, CMR, GCK and PM treatments, respectively. Among the most frequent genera, only 10, including *Gemmatimonas*, *Gp1*, *Gp4*, *Gp6*, *Burkholderia*, *Gp3*, *Nitrospira*, *Ohtaekwangia*, *TM7_genus_incertae_sedis* and *3_genus_incertae_sedis* were represented in all treatments. Moreover, in comparison to other treatments, significantly higher abundances of the genera *Gemmatimonas* and *Gp4* were observed in BIO-treated soil among the most 10 abundant genera.

**Table 2:** Frequency of the most abundant bacterial genera, indicated in % of all classified sequences, within each treatment of bio-organic fertilizer (BIO), cattle manure compost (CM), Chinese medicine residue compost (CMR), general operation control (GCK) and pig manure compost (PM).

%	BIO	CM	CMR	GCK	PM	Phylum
*Gemmatimonas*	5.56±0.42a	3.62±0.22c	3.27±0.25c	4.38±0.25b	3.33±0.10c	*Gemmatimonadetes*
*Gp1*	5.49±0.31c	6.54±0.17b	7.07±0.39a	2.43±0.07d	6.59±0.75b	*Acidobacteria*
*Gp4*	4.62±0.27a	2.15±0.08d	2.19±0.35d	3.55±0.46b	2.72±0.14c	*Acidobacteria*
*Gp6*	4.49±0.19a	1.49±0.07c	2.38±0.16b	5.31±1.07a	2.73±0.60b	*Acidobacteria*
*Burkholderia*	3.76±1.00d	8.68±0.77b	10.79±2.02a	1.46±0.43e	6.51±1.90c	*Proteobacteria*
*Gp3*	2.90±0.19a	2.84±0.45a	2.10±0.23b	2.35±0.68a	2.77±0.10a	*Acidobacteria*
*Nitrospira*	2.64±0.10b	2.07±0.01c	1.66±0.23d	2.73±0.12b	3.01±0.17a	*Nitrospirae*
*Ohtaekwangia*	1.70±0.19d	2.18±0.13c	1.32±0.06e	3.31±0.11a	2.83±0.16b	*Bacteroidetes*
*TM7_genus_incertae_sedis*	1.55±0.09b	2.54±0.04a	2.52±0.11a	1.33±0.30b	2.44±0.38a	*TM7*
*3_genus_incertae_sedis*	1.07±0.19a	1.13±0.12a	1.11±0.07a	1.17±0.42a	1.39±0.10a	*Verrucomicrobia*
*Sphingomonas*	1.71±0.49a	1.10±0.05b	1.47±0.05a			*Proteobacteria*
*Gp5*	1.17±0.12a			1.12±0.12a	1.12±0.17a	*Acidobacteria*
*Bacillus*		1.67±0.11a	1.78±0.06a		1.44±0.12b	*Firmicutes*
*Niastella*		2.96±0.23a			1.55±0.20b	*Bacteroidetes*
*Gp2*		1.48±0.19b	1.09±0.05c		1.68±0.05a	*Acidobacteria*
*Beggiatoa*				1.46±0.16		*Proteobacteria*
*Gp13*					1.49±0.06	*Acidobacteria*
*Segetibacter*		1.87±0.12				*Bacteroidetes*
*Chitinophaga*		1.36±0.04				*Bacteroidetes*
*Frateuria*			1.06±0.13			*Proteobacteria*

Only the genera frequency higher than 1% was listed in the table. Values are the means followed by standard error of the mean. Different letters indicate statistically significant differences at the 0.05 probability level according to Fisher's least significant difference test (LSD) and the Duncan test.

### Bacterial α-diversity

The bacterial richness and diversity of the different fertilizer treatments were calculated based on 7,817 randomly selected sequences (Table 3). The richness index, Chao1 and ACE showed that the CM-treated soil exhibited the lowest number of OTUs, while the BIO-treated soil showed the highest number with no significant difference between the CMR, PM and GCK treatments. The CM treatment had the lowest Shannon diversity index value (*H*′), while the highest values were of the GCK and PM treatments. CM treatment showed the highest Good's query Coverage (ranging from 0.87 to 0.90 for all treatments), and no significant difference was observed for the other treatments.

**Table 3:** Calculations of Chao1, ACE, Shannon and Good's Coverage indices for treatments with bio-organic fertilizer (BIO), cattle manure compost (CM), Chinese medicine residue compost (CMR), general operation control (GCK) and pig manure compost (PM) at a 97% similarity threshold.

Treatments	Chao1	ACE	Shannon	Coverage
BIO	3,751±220a	5,398±292a	6.60±0.04b	0.87±0.01b
CM	3,105±75b	4,085±91b	6.38±0.05c	0.90±0.01a
CMR	3,477±174a	4,904±216ab	6.46±0.03c	0.88±0.01b
GCK	3,588±173a	5,112±395a	6.76±0.05a	0.88±0.01b
PM	3,724±236a	5,573±108a	6.70±0.04a	0.88±0.01b

Values indicate the means followed by standard error of the mean. Different letters indicate statistically significant differences at the 0.05 probability level according to Fisher's least significant difference test (LSD) and the Duncan test.

**Figure 2:**
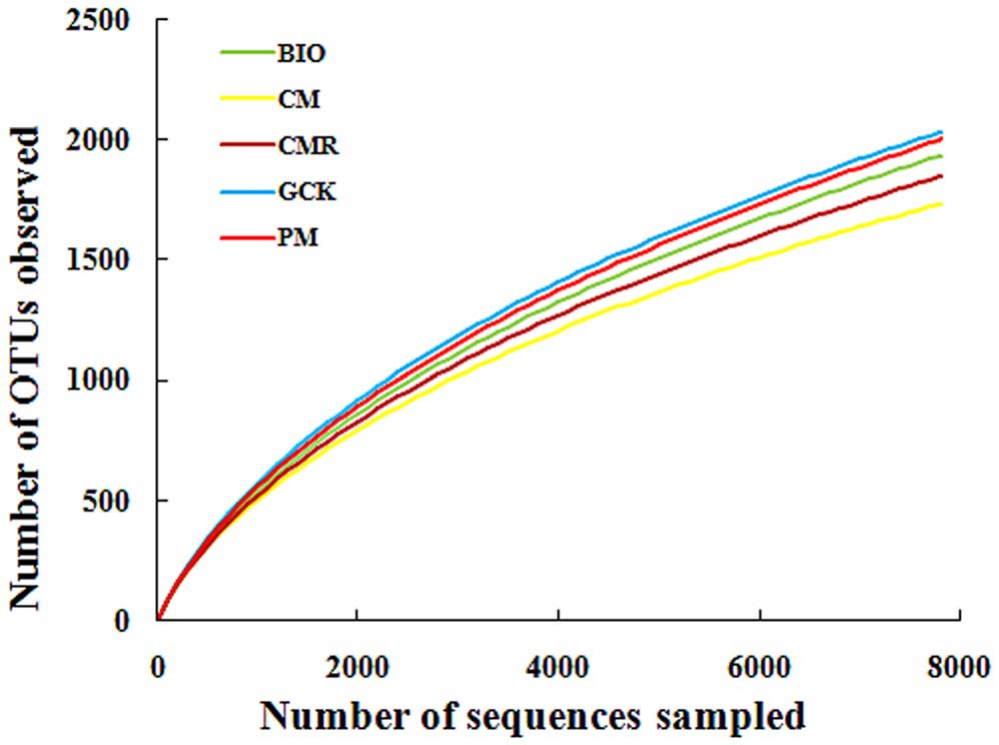
Rarefaction analysis at different 3% dissimilarity levels for treatments with bio-organic fertilizer (BIO), cattle manure compost (CM), Chinese medicine residue compost (CMR), general operation control (GCK) and pig manure compost (PM).

Similar results were observed with 3% dissimilarity after comparing the rarefaction curves of the mean pooled sequences of 3 replicates of each treatment, with the GCK treatment showing the highest OTU number and CM treatment showing the lowest OTU number, However, the rarefaction curves did not reach saturation, which indicated that more sequencing efforts were needed (Fig. 2).

### Bacterial community structure

**Figure 3:**
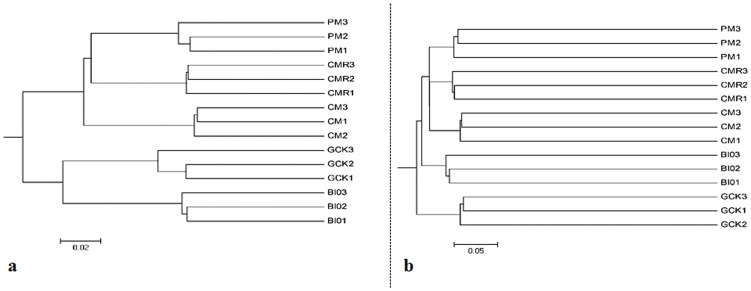
Hierarchical cluster tree constructed based on the distance matrix that was calculated using the (a) weighted UniFrac algorithm and (b) unweighted UniFrac algorithm for treatments with bio-organic fertilizer (BIO), cattle manure compost (CM), Chinese medicine residue compost (CMR), general operation control (GCK) and pig manure compost (PM).

The analysis of microbial communities using hierarchical cluster analysis showed that the bacterial communities from the same treatment were more similar to each other than those from different treatments, as observed for the 5 highly supported clusters that were made up of samples from different fertilizer-treated soils (Fig. 3). Bacterial community structure from soil samples that were amended with common composts (CM, CMR, and PM) clustered together while soil samples from BIO and GCK were clustered together based on weighted UniFrac algorithm (Fig. 3a). Bacterial community membership from soil samples that were amended with organic amendments (CM, CMR, PM and BIO) clustered together and were separated to general operation control (GCK) based on unweighted UniFrac algorithm (Fig. 3b). Moreover, BIO-treated soil grouped separately from common compost treatments (CM, CMR and PM), which were grouped together.

**Figure 4:**
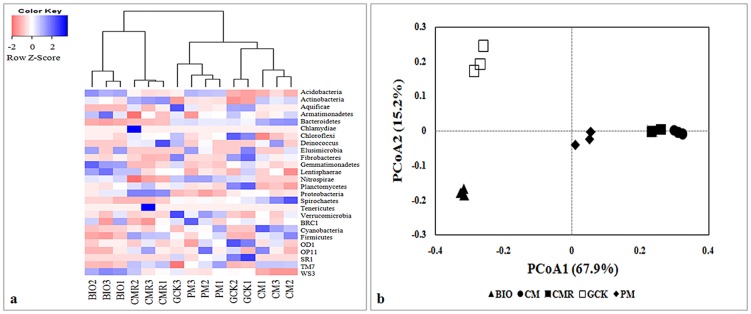
Heat map of the bacterial communities based on abundance of phyla (a) and Jackknifed principal coordination analysis (PCoA) plots with unweighted UniFrac distance metric (b) from treatments with bio-organic fertilizer (BIO), cattle manure compost (CM), Chinese medicine residue compost (CMR), general operation control (GCK) and pig manure compost (PM). Color from pink to blue indicates increasing abundance.

Heat map analysis of the abundant phyla within a hierarchical cluster based on Bray–Curtis distance indices showed different patterns of community structure among the different treatments and similar patterns for the same treatment in triplicate (Fig. 4a). Moreover, BIO treatment showed a different pattern of community structure from those of other soil samples and enriched phyla of *Acidobacteria*, *Gemmatimonadetes*, *WS3* and *Lentisphaerae*, as shown in blue. Principal coordinates analysis (PCoA) based on the OTU composition also clearly showed variations among these different fertilizer treatments (Fig. 4b). The first two principal components could explain 83.1% of the variation of the individual samples of the total bacterial community. The bacterial community of the BIO-treated soil was well-separated from that of common compost-treated soils (CM, PM and CMR) along the first component (PCoA1) and was separated from the general control (GCK) along the second component (PCoA2).

### Relationship between disease incidence and the selected parameters

**Figure 5:**
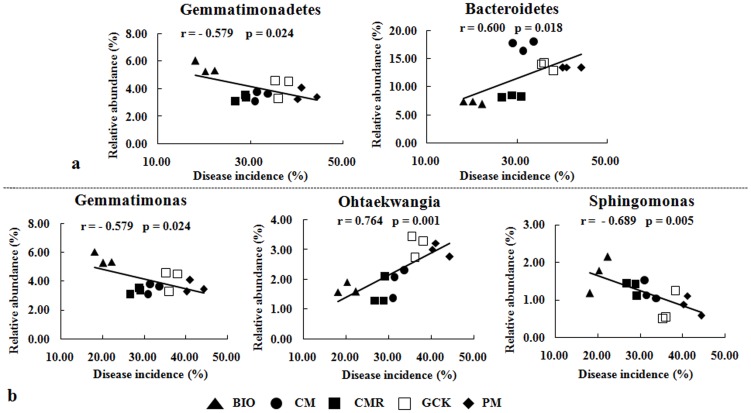
Correlation analysis between the relative abundance of two bacteria phyla (a), three of the most classified bacteria genera (b) and banana *Fusarium* wilt disease incidence for treatments with bio-organic fertilizer (BIO), cattle manure compost (CM), Chinese medicine residue compost (CMR), general operation control (GCK) and pig manure compost (PM).

According to the disease incidence reported in our previous paper [12] and based on line regression analysis, a significant correlation between the abundance of the *Gemmatimonadetes*, *Bacteroidetes*, *Lentisphaerae* and *SR1* phyla and *Fusarium* wilt disease incidence was found (Table S2). Among these phyla, *Lentisphaerae* and *SR1* were not considered further due to their low abundance and random distribution. A clear negative correlation between *Gemmatimonadetes* (r = −0.579, p = 0.024) and the disease incidence and a clear positive correlation between *Bacteroidetes* (r = 0.600, p = 0.018) and the disease incidence were observed (Fig. 5a).

Line regression analysis between the 20 most-abundant classified genera and disease incidence showed that *Gemmatimonas*, *Ohtaekwangia* and *Sphingomonas* were significantly correlated to disease incidence (Table S3). A strong negative correlation between disease incidence and *Gemmatimonas* (r = −0.579, p = 0.024) and *Sphingomonas* (r = −0.689, p = 0.005) and a positive correlation with *Ohtaekwangia* (r = 0.764, p = 0.001) were observed (Fig. 5b). Unfortunately, some classified genera that were generally considered to contain plant growth-promoting rhizobacteria (PGPR) strains, which can suppress soil-borne fungi or promote plant growth, were only present in limited amounts, and their presence was not correlated with disease incidence (Table S4). Furthermore, in our research, no significant correlation was found between the whole bacteria community indices (richness and diversity) and disease incidence (Table S5).

**Figure 6:**
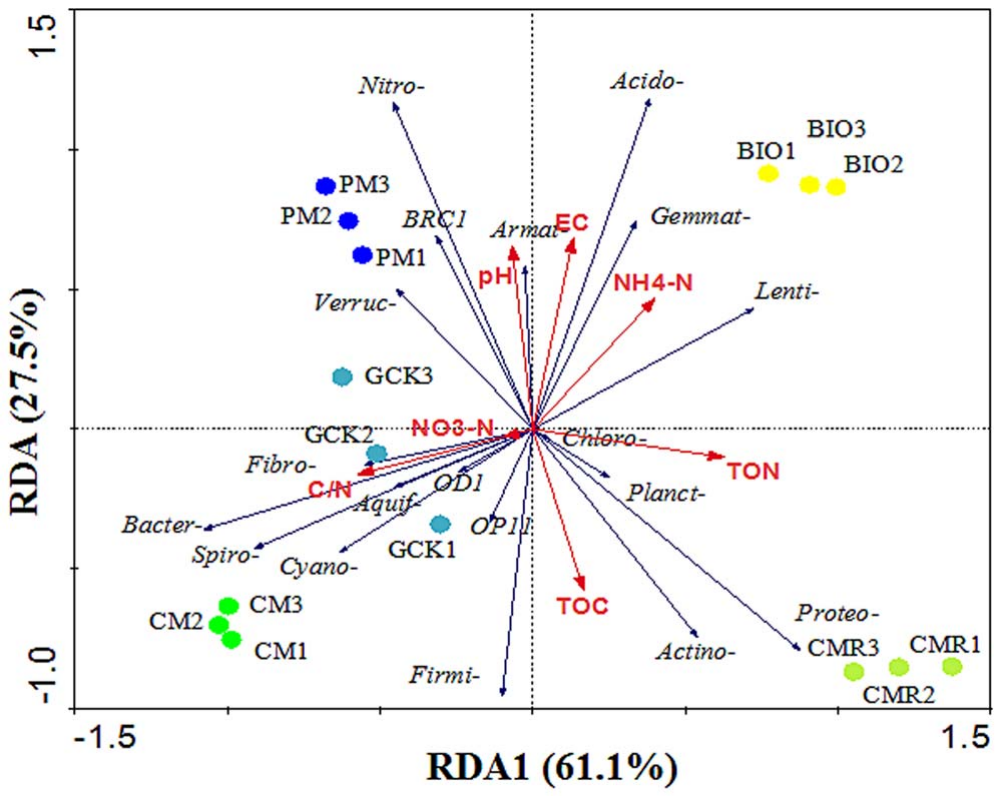
Redundancy analysis (RDA) of the abundant phyla and soil properties for soil samples from treatments with bio-organic fertilizer (BIO), cattle manure compost (CM), Chinese medicine residue compost (CMR), general operation control (GCK) and pig manure compost (PM).

The RDA that was performed on the phyla data and soil chemical properties showed that the first two RDA components could explain 88.6% of the total variation (Fig. 6). The first component (RDA1) separated the BIO and CMR treatments from the other fertilizer treatments and explained 61.1% of the variation, and the second component (RDA2), which separated the BIO from the CMR treatment, explained 27.5% of the variation. All soil chemical properties sufficiently explained the variation in phyla data (p = 0.002, Monte Carlo test). Ammonium nitrogen (NH4-N) and electricity conductivity (EC) accounted for a large amount of the variation in the distribution of the BIO treatment from other treatments along the RDA1 and RDA2 axes. As shown by their close grouping and by the vectors, BIO-treated soil with the lowest disease incidence was positively related to the higher relative abundant phyla of *Gemmatimonadetes* and *Lentisphaerae*, the higher content of NH4-N and the EC, and it was negatively related to *Bacteroidetes*, a higher content of soil nitrate nitrogen (NO3-N) and higher total carbon to nitrogen ratio (C/N). Furthermore, the relative abundance of *Gemmatimonadetes* was positively correlated with soil pH, EC and NH4-N contents and negatively correlated with the soil total carbon (TOC) and C/N ratio. Moreover, the relative abundance of *Lentisphaerae* was positively correlated with the total nitrogen (TON) and NH4-N contents of the soil and negatively correlated with the soil C/N ratio. In contrast, the relative abundance of *Bacteroidetes* was positively correlated with the soil C/N ratio and negatively correlated with the soil TON and NH4-N contents (Fig. 6 and Table S6).

## Discussion

In our previous study, the main potential mechanism by which the BIO application reduced the *Fusarium* population has been revealed by culture-depended and PCR-DGGE methods [12]. However, deeper research should be done to further explore the potential mechanism. To our knowledge, this detailed comparison of the soil bacteria community after the application of BIO or other common composts in a banana orchard with serious *Fusarium* wilt disease was the first to be assessed using deep 16 S rRNA pyrosequencing, although this method has been used to study the long-term effects of selected, common composts on the soil bacteria community composition or structure [15], [36]. The obtained results supported the hypothesis that soil amended with different organic materials showed different responses by the bacterial community or suppression of *Fusarium* wilt disease [15], [37]–[39].

Phyla analysis revealed that *Proteobacteria*, *Acidobacteria*, *Bacteroidetes*, *Gemmatimonadetes*, *Actinobacteria* and *Firmicutes* were the most common phyla, but with some variety in relative abundance. This finding roughly corresponded with those of previous articles that investigated agricultural or other type soils in which these phyla accounted for more than 74.0% of the sequences that were examined using deep 16 S rRNA pyrosequencing [18], [19]. The relative abundance of *Acidobacteria* was relatively high in our study due to the experiment being conducted in acidic soil [40], [41]. However, in our study, BIO and PM treatments with the higher pH showed the higher relative abundance of *Acidobacteria*. This finding was contrary to a previous study that showed that pH had a negative relation to *Acidobacteria* abundance [40], [42]. The reason for this phenomenon is still unclear and may be due to the narrow pH value range of the treated soil; however, a few articles have shown no obvious correlation between pH and abundance of *Acidobacteria* [15], [36]. Analysis of the most abundant genera (>1%) also revealed significant differences between the bacterial communities of different treatments, a higher abundance of *Gemmatimonas* and *Gp4* in BIO-treated soil compared to other soil samples.

These changes could correspond to the decline of *Fusarium* wilt disease incidence. Thus, further correlation analysis was performed. Interestingly, the results showed that *Fusarium* wilt disease incidence might be related to the *Gemmatimonadetes* and *Bacteroidetes* phyla and/or *Gemmatimonas* genus, which belongs to *Gemmatimonadetes*, *Ohtaekwangia*, which belongs to *Bacteroidetes*, and *Sphingomonas*, which belongs to *Proteobacteria*. The high abundance of the *Bacteroidetes* phylum and the *Ohtaekwangia* genus that was observed in this study might positively correspond to *Fusarium* wilt disease incidence because this finding is in accordance with the report that the relative abundance of *Bacteroidetes* was similar between the initial and disease stages and followed by a significant decrease when suppressiveness was reached, as investigated using a 16 S rRNA-based microarray method [43], although, *Bacteroidetes* was also reported to possess the potential ability for biocontrol [44]. Moreover, we found the *Gemmatimonadetes* phylum and *Gemmatimonas* and *Sphingomonas* genera might respond to the suppression of *Fusarium* wilt disease via BIO application. *Gemmatimonas* and *Gemmatimonadetes* are a recently proposed genus and phylum, respectively, and they widely exist in multiple terrestrial and aquatic habitats. However, little is known about the ecological functions of this genus/phylum, except that Yin et al. [45] reported that the *Gemmatimonas* genus was found at a higher frequency in the rhizosphere of healthy plants using 454 pyrosequencing. *Sphingomonas*, which belongs to the *Sphingomonadaceae* order and *Proteobacteria* phylum, is widely distributed in natural habitats and is utilized for a wide range of biotechnological applications due to its remarkable biodegradative and biosynthetic capabilities [46]. Kyselková et al. [44] reported that bacteria affiliated with *Sphingomonadaceae* were more prevalent in tobacco-suppressive rhizosphere soil. Wachowska et al. [47] also reported that *Sphingomonas* could be used as biological agents to control winter wheat pathogens, such as *Fusarium*, under greenhouse conditions.

Analysis using rarefaction, Chao1 and ACE showed that the OTU numbers for BIO treatment were not significantly higher than for the other treatments. Furthermore, the diversity for BIO treatment that was estimated by the Shannon index and Coverage was also not the highest. All of the results indicated that a 1-year application of BIO could not significantly increase the bacteria community richness and diversity at the whole-community-structure level, which was in accordance with results of a previous study that used pyrosequencing to show that soil bacterial community richness and diversity were similar after a 5-year application of different organic amendments [15]. Although many previous articles indicated that the richness and/or diversity of the soil microbial community may respond to disease incidence [12], [38], this phenomenon was not observed in this study because no obvious correlation between the indices and *Fusarium* wilt disease was observed (Table S5). This may be due to all 1-year treatments being performed on the same soil, which possessed similar bacteria community indices at the beginning.

In our study, the results of phylogenetic structure analyzed using the hierarchical cluster tree, heat map analysis based on the phyla frequency and PCoA analysis based on the OTU composition all showed that the bacterial community of BIO-treated soil differed from the common compost treatments (CM, CMR, and PM) and the control (GCK). All of the results confirmed that BIO application altered the bacterial community, which was roughly similar to the results of our previous investigation using PCR-DGGE that showed that BIO-treated soil grouped away from other soil samples [12]. Poulsen et al. [15] also reported similar results suggesting that soil amended with MSW-compost was separate from other amendments or the control, which indicated that the soil bacterial community responds differently to different compost amendments.

It has been reported that the chemical properties of soil can influence the suppressiveness of soil towards diseases [48]. In our RDA analysis, the BIO treatment with lowest *Fusarium* wilt disease incidence was highly correlated with the highest proportion of *Gemmatimonadetes* and lowest proportion of *Bacteroidetes*. Furthermore, the proportion of *Gemmatimonadetes* was positively correlated with soil pH, EC and NH4-N and negatively correlated with TOC and the C/N ratio. However, *Bacteroidetes* was positively correlated with the soil C/N ratio and negatively correlated with TON and NH4-N (Fig. 5, Table S6). Therefore, suppression of *Fusarium* wilt disease might be highly correlated with soil properties because *Fusarium* wilt disease incidence was positively correlated with the C/N ratio and negatively correlated to NH4-N and TON (Table S7), which was in agreement with reports from several previous studies. For example, Hamel et al. [49] reported a positive association between the TON content of the soil and the suppressiveness towards *Fusarium* spp. on asparagus. However, the form of N, either as NO3-N or NH4-N, is also important for disease suppression. Pérez-Piqueres et al. [50] reported that suppressive soil contained higher rates of NH4–N than conductive soil when studying the effect of compost amendment on soil suppressiveness toward *Rhizoctonia solani* disease, and Mallett and Maynard [51] reported that the incidence of *Armillaria* root disease significantly increased with decreasing NH4-N concentration on the organic surface horizon. In contrast, Oyarzun et al. [52] reported that the disease suppression ability of *Thielaviopsis basicola* was positively associated with a decreased C/N ratio.

In this study, after analyzing all of the data, the abundance of *Bacillus* was not enriched after BIO application. This finding combined with our previous results, the main mechanism reduced the *Fusarium* population for BIO application might be attributed to a general suppression that the BIO application altered the soil microbial composition and stimulated the population of soil bacteria, actinomycetes and some beneficial microorganisms [12], indicated that the genus might not necessarily reflect the individual species that has functional importance in suppressing endemic soil disease and all the results revealed by further deep 16S rRNA pyrosequencing confirmed that the main potential mechanism by which the BIO application reduced the *Fusarium* population was deduced to the fact that the specific bio-organic fertilizer containing functional microbes altered the soil microbial composition and stimulated the population of some beneficial microorganisms, thus resulting in a general suppression.

## Conclusions

Deep 16 S rRNA pyrosequencing assessment of soil bacterial communities from different compost-treated soil in a monoculture banana orchard revealed significant differences among all treatments, including differences in community structure, composition, richness, diversity and bacterial phylogeny. Phyla of *Gemmatimonadetes* and *Acidobacteria* were significantly elevated in BIO treatment in comparison to other treatments. A decrease was also found for *Bacteroidetes* in BIO treatment. Moreover, genera of *Gemmatimonas* and *Gp4* were significantly elevated in BIO treatment in comparison to other treatments. Additionally, the enrichment of *Gemmatimonas* and *Sphingomonas* and the TON and NH4-N soil content was positively correlated with disease suppression. Cumulatively, the reduction of the *Fusarium* wilt disease incidence after a 1-year application of BIO might be attributed to the fact that application of a BIO fertilizer containing *Bacillus* sp. induced general suppression in the soil by modulating the bacterial community and specific suppression by enriching *Gemmatimonas* and *Sphingomonas*.

## Supporting Information

Table S1 **Primer sequences used for preparation of samples for deep 16S rRNA pyrosequencing.**
(DOCX)

Table S2 **Line regression coefficient of the most abundant phyla (>1%) and Fusarium wilt disease incidence.** * in the table means correlation is significant at the 0.05 level, ** in the table means correlation is significant at the 0.01 level.
(DOCX)

Table S3 **Line regression coefficient of the most frequent classified genera (>1%) and Fusarium wilt disease incidence.** * in the table means correlation is significant at the 0.05 level, ** in the table means correlation is significant at the 0.01 level.
(DOCX)

Table S4 **Line regression coefficient of selected bacteria genera and Fusarium wilt disease incidence.** * in the table means correlation is significant at the 0.05 level, ** in the table means correlation is significant at the 0.01 level.
(DOCX)

Table S5 **Line regression coefficient of the bacteria community indices and Fusarium wilt disease incidence.** * in the table means correlation is significant at the 0.05 level, ** in the table means correlation is significant at the 0.01 level.
(DOCX)

Table S6 **Line regression coefficient (r) between selected phyla in all samples and soil properties.** * in the table means correlation is significant at the 0.05 level, ** in the table means correlation is significant at the 0.01 level.
(DOCX)

Table S7 **Line regression coefficient (r) between Fusarium wilt disease incidence in all samples and soil properties.** * in the table means correlation is significant at the 0.05 level, ** in the table means correlation is significant at the 0.01 level.
(DOCX)
