# Supplementary material for: Deep 16S rRNA Pyrosequencing Reveals a Bacterial Community Associated with Banana Fusarium Wilt Disease Suppression Induced by Bio-Organic Fertilizer Application
Source: PLoS One. 2014 May 28;9(5):e98420. doi: 10.1371/journal.pone.0098420 (PMC4037203; doi:10.1371/journal.pone.0098420)
Supplement: Table S1 — Primer sequences used for preparation of samples for deep 16S rRNA pyrosequencing. (DOCX) [file pone.0098420.s001.docx]

**Table S1**

| Name | Sequence | Sample name |
| --- | --- | --- |
| Adapter A | CGTATCGCCTCCCTCGCGCCATCAG |  |
| Linker A | TC |  |
| Forward primer-27F | AGAGTTTGATCCTGGCTCAG |  |
| Adapter B | CTATGCGCCTTGCCAGCCCGCTCAG |  |
| Linker B | AC |  |
| Reverse primer-533R | TTACCGCGGCTGCTGGCAC |  |
| barcode | AGATACGCTG | PM1 |
| barcode | AGATCTAGTC | PM2 |
| barcode | AGCAGCGTAG | PM3 |
| barcode | AGCGCACGAG | CM1 |
| barcode | AGCGTGTGCG | CM2 |
| barcode | AGCTAGATAC | CM3 |
| barcode | AGCTGTCGAC | GCK1 |
| barcode | AGTATGCACG | GCK2 |
| barcode | AGTCGCGCTA | GCK3 |
| barcode | AGTCTGTCTG | BIO1 |
| barcode | ATACACACGA | BIO2 |
| barcode | ATACGCGTGC | BIO3 |
| barcode | ATACTAGCAC | CMR1 |
| barcode | ATAGAGCTAG | CMR2 |
| barcode | ATATAGAGTA | CMR3 |
