# Supplementary material for: Deep 16S rRNA Pyrosequencing Reveals a Bacterial Community Associated with Banana Fusarium Wilt Disease Suppression Induced by Bio-Organic Fertilizer Application
Source: PLoS One. 2014 May 28;9(5):e98420. doi: 10.1371/journal.pone.0098420 (PMC4037203; doi:10.1371/journal.pone.0098420)
Supplement: Table S2 — Line regression coefficient of the most abundant phyla (>1%) and Fusarium wilt disease incidence. * in the table means correlation is significant at the 0.05 level, ** in the table means correlation is significant at the 0.01 level. (DOCX) [file pone.0098420.s002.docx]

**Table S2**

| Most abundant phylum | r | p-value |
| --- | --- | --- |
| *Proteobacteria* | -0.48 | 0.07 |
| *Acidobacteria* | -0.36 | 0.19 |
| *Bacteroidetes* | 0.60* | 0.02 |
| *Gemmatimonadetes* | -0.58* | 0.02 |
| *Actinobacteria* | -0.48 | 0.07 |
| *Firmicutes* | 0.19 | 0.53 |
| *Nitrospirae* | 0.34 | 0.21 |
| *TM7* | 0.18 | 0.53 |
| *Chloroflexi* | 0.42 | 0.18 |
| *Verrucomicrobia* | 0.35 | 0.20 |
| *Planctomycetes* | 0.24 | 0.40 |
| *Armatimonadetes* | -0.25 | 0.37 |
| *OD1* | 0.44 | 0.11 |
| *WS3* | -0.50 | 0.06 |
| *Fibrobacteres* | 0.41 | 0.13 |
| *Cyanobacteria* | 0.20 | 0.48 |
| *BRC1* | 0.13 | 0.66 |
| *Lentisphaerae* | -0.62** | 0.01 |
| *Spirochaetes* | 0.33 | 0.23 |
| *SR1* | 0.56* | 0.03 |
| *OP11* | 0.35 | 0.21 |
