# Supplementary material for: Deep 16S rRNA Pyrosequencing Reveals a Bacterial Community Associated with Banana Fusarium Wilt Disease Suppression Induced by Bio-Organic Fertilizer Application
Source: PLoS One. 2014 May 28;9(5):e98420. doi: 10.1371/journal.pone.0098420 (PMC4037203; doi:10.1371/journal.pone.0098420)
Supplement: Table S3 — Line regression coefficient of the most frequent classified genera (>1%) and Fusarium wilt disease incidence. * in the table means correlation is significant at the 0.05 level, ** in the table means correlation is significant at the 0.01 level. (DOCX) [file pone.0098420.s003.docx]

**Table S3**

| Most classified genera | r | p-value |
| --- | --- | --- |
| *Gemmatimonas* | -0.58* | 0.02 |
| *Gp1* | -0.22 | 0.43 |
| *Gp4* | -0.46 | 0.09 |
| *Gp6* | -0.09 | 0.76 |
| *Burkholderia* | -0.18 | 0.52 |
| *Gp3* | -0.06 | 0.81 |
| *Nitrospira* | 0.34 | 0.21 |
| *Ohtaekwangia* | 0.76** | 0.00 |
| *TM7_genus_incertae_sedis* | 0.18 | 0.53 |
| *3_genus_incertae_sedis* | 0.37 | 0.18 |
| *Sphingomonas* | -0.69** | 0.01 |
| *Gp5* | 0.13 | 0.66 |
| *Bacillus* | 0.09 | 0.74 |
| *Niastella* | 0.11 | 0.69 |
| *Gp2* | 0.38 | 0.16 |
| *Beggiatoa* | 0.21 | 0.46 |
| *Gp13* | 0.47 | 0.07 |
| *Segetibacter* | 0.17 | 0.54 |
| *Chitinophaga* | -0.05 | 0.87 |
| *Frateuria* | -0.07 | 0.81 |
