# Supplementary material for: Deep 16S rRNA Pyrosequencing Reveals a Bacterial Community Associated with Banana Fusarium Wilt Disease Suppression Induced by Bio-Organic Fertilizer Application
Source: PLoS One. 2014 May 28;9(5):e98420. doi: 10.1371/journal.pone.0098420 (PMC4037203; doi:10.1371/journal.pone.0098420)
Supplement: Table S4 — Line regression coefficient of selected bacteria genera and Fusarium wilt disease incidence. * in the table means correlation is significant at the 0.05 level, ** in the table means correlation is significant at the 0.01 level. (DOCX) [file pone.0098420.s004.docx]

**Table S4**

| Selected PGPR groups | r | p-value |
| --- | --- | --- |
| *Burkholderia* | -0.18 | 0.52 |
| *Bacillus* | 0.09 | 0.74 |
| *Pseudomonas* | 0.39 | 0.15 |
| *Mycobacterium* | -0.21 | 0.45 |
| *Acinetobacter* | 0.04 | 0.88 |
| *Legionella* | 0.42 | 0.12 |
| *Nocardia* | 0.18 | 0.53 |
| *Streptomyces* | -0.27 | 0.39 |
| *Bradyrhizobium* | 0.00 | 0.99 |
| *Arthrobacter* | -0.09 | 0.74 |
| *Herbaspirillum* | -0.30 | 0.27 |
| *Thiobacillus* | -0.49 | 0.07 |
