# Supplementary material for: Deep 16S rRNA Pyrosequencing Reveals a Bacterial Community Associated with Banana Fusarium Wilt Disease Suppression Induced by Bio-Organic Fertilizer Application
Source: PLoS One. 2014 May 28;9(5):e98420. doi: 10.1371/journal.pone.0098420 (PMC4037203; doi:10.1371/journal.pone.0098420)
Supplement: Table S5 — Line regression coefficient of the bacteria community indices and Fusarium wilt disease incidence. * in the table means correlation is significant at the 0.05 level, ** in the table means correlation is significant at the 0.01 level. (DOCX) [file pone.0098420.s005.docx]

**Table S5**

| Index | r | p-value |
| --- | --- | --- |
| Chao1 | -0.07 | 0.81 |
| ACE | 0.06 | 0.82 |
| Shannon | 0.44 | 0.10 |
| Coverage | 0.20 | 0.49 |
