# Supplementary material for: Deep 16S rRNA Pyrosequencing Reveals a Bacterial Community Associated with Banana Fusarium Wilt Disease Suppression Induced by Bio-Organic Fertilizer Application
Source: PLoS One. 2014 May 28;9(5):e98420. doi: 10.1371/journal.pone.0098420 (PMC4037203; doi:10.1371/journal.pone.0098420)
Supplement: Table S6 — Line regression coefficient (r) between selected phyla in all samples and soil properties. * in the table means correlation is significant at the 0.05 level, ** in the table means correlation is significant at the 0.01 level. (DOCX) [file pone.0098420.s006.docx]

**Table S6**

| Treatments | pH | EC | TOC | TON | C/N | NH4-N | NO3-N |
| --- | --- | --- | --- | --- | --- | --- | --- |
| *Bacteroidetes* | -0.18 | -0.41 | 0.10 | -0.69** | 0.70** | -0.63* | 0.16 |
| *Gemmatimonadetes* | 0.77** | 0.56* | -0.53* | 0.60 | -0.85** | 0.89** | 0.24 |
| *Lentisphaerae* | 0.41 | 0.42 | -0.23 | 0.68** | -0.77** | 0.74** | -0.11 |
