# Supplementary material for: Deep 16S rRNA Pyrosequencing Reveals a Bacterial Community Associated with Banana Fusarium Wilt Disease Suppression Induced by Bio-Organic Fertilizer Application
Source: PLoS One. 2014 May 28;9(5):e98420. doi: 10.1371/journal.pone.0098420 (PMC4037203; doi:10.1371/journal.pone.0098420)
Supplement: Table S7 — Line regression coefficient (r) between Fusarium wilt disease incidence in all samples and soil properties. * in the table means correlation is significant at the 0.05 level, ** in the table means correlation is significant at the 0.01 level. (DOCX) [file pone.0098420.s007.docx]

**Table S7**

|  | pH | EC | TOC | TON | C/N | NH_4_-N | NO_3_-N |
| --- | --- | --- | --- | --- | --- | --- | --- |
| Disease incidence | -0.22 | -0.04 | 0.25 | -0.62* | 0.82** | -0.57* | -0.492 |
